# The Scientific and Clinical Case for Reviewing Diagnostic Radiopharmaceutical Extravasation Long-Standing Assumptions

**DOI:** 10.3389/fmed.2021.684157

**Published:** 2021-06-28

**Authors:** Dustin Osborne, Ronald Lattanze, Josh Knowland, Tonia E. Bryant, Iryna Barvi, Yitong Fu, Jackson W. Kiser

**Affiliations:** ^1^Radiology Department, University of Tennessee Graduate School of Medicine, Knoxville, TN, United States; ^2^Lucerno Dynamics LLC, Cary, NC, United States; ^3^Department of Molecular Imaging, Carilion Clinic, Roanoke, VA, United States

**Keywords:** extravasation, infiltration, patient harm, adverse events, quality, dosimetry

## Abstract

**Background:** The patient benefit from a diagnostic nuclear medicine procedure far outweighs the associated radiation risk. This benefit/risk ratio assumes a properly administered radiopharmaceutical. However, a significant diagnostic radiopharmaceutical extravasation can confound the procedure in many ways. We identified three current extravasation hypotheses espoused by medical societies, advisory committees, and hundreds of individual members of the nuclear medicine community: diagnostic extravasations do not cause harm, do not result in high absorbed dose to tissue, and require complex dosimetry methods that are not readily available in nuclear medicine centers. We tested these hypotheses against a framework of current knowledge, recent developments, and original research. We conducted a literature review, searched regulatory databases, examined five clinical cases of extravasated patients, and performed dosimetry on those extravasations to test these globally accepted hypotheses.

**Results:** A literature review found 58 peer-reviewed documents suggesting patient harm. Adverse event/vigilance report database reviews for extravasations were conducted and revealed 38 adverse events which listed diagnostic radiopharmaceutical extravasation as a factor, despite a regulatory exemption for required reporting. In our own case material, assessment of care was evaluated for five extravasated patients who underwent repeat imaging. Findings reflected results of literature review and included mis- or non-identification of lesions, underestimation of Standardized Uptake Values (SUVs) by 19–73%, classification of scans as non-diagnostic, and the need to repeat imaging with the associated additional radiation exposure, inconvenience, or delays in care. Dosimetry was performed for the same five cases of diagnostic radiopharmaceutical extravasation. Absorbed doses to 5 cm^3^ of tissue were between 1.1 and 8.7 Gy, and shallow dose equivalent for 10 cm^2^ of skin was as high as 4.2 Sv.

**Conclusions:** Our findings suggest that significant extravasations can or have caused patient harm and can irradiate patients' tissue with doses that exceed medical event reporting limits and deterministic effect thresholds. Therefore, diagnostic radiopharmaceutical injections should be monitored, and dosimetry of extravasated tissue should be performed in certain cases where thresholds are thought to have been exceeded. Process improvement efforts should be implemented to reduce the frequency of extravasation in nuclear medicine.

## Introduction

Diagnostic nuclear medicine procedures have been consistently and appropriately viewed through the lens that the benefits from these procedures demonstrably exceed the inherent radiation risk of the procedure. In 2002, fifty nuclear medicine leaders emphasized in the Guide for Diagnostic Nuclear Medicine (1) “more than one-third of a billion doses have been administered to patients, with a track record for safety that is unparalleled. The radiation doses associated with diagnostic nuclear medicine procedures average 4.4 mSv effective dose equivalent according to a National Council on Radiation Protection and Measurement study published in 1991.” The authors also describe that deterministic effects can “occur only after relatively high dose levels that exceed the threshold for those effects, usually a dose on the order of 100 roentgen equivalent man (rem) (1 Sv)” and “the risk of stochastic effects increases as a function of radiation dose.” They state that “the risk of deterministic effects attributed to the exposures likely to be encountered in diagnostic nuclear medicine procedures is insignificant.” In diagnostic nuclear medicine procedures where the radiopharmaceutical is assumed to be injected properly, the absorbed dose to patient tissue would be <1 mGy or 1 mSv dose equivalent.

An infiltration is the inadvertent injection of a pharmaceutical into the tissue instead of the vein, as intended. Although an extravasation is typically defined as an infiltration of a vesicant (2), an infiltration of a radiopharmaceutical can be considered an extravasation due to the effects of ionizing radiation on patient tissue. In 2020, the Nuclear Regulatory Commission (NRC) which regulates the use of radioactive isotopes in the United States requested public comments on a petition that the NRC eliminate a policy. The policy exempts extravasations from medical event reporting, even if they meet reporting requirements. The NRC's Advisory Committee on the Medical Uses of Isotopes (ACMUI), medical societies, and leading members of the nuclear medicine and radiology communities have provided their position on diagnostic extravasations to the NRC.

In a September 2019 meeting with the NRC regarding extravasations, ACMUI members reported they were “unaware of any cases where there has been patient harm due to extravasation” (3). The public comments submitted to the NRC regarding the extravasation petition also support the commonly held hypothesis. In 396 submitted comments, medical societies or individual members stated there is no clinical data that diagnostic radiopharmaceutical extravasation is a patient safety issue. Other comments provide additional insight into these hypotheses regarding diagnostic extravasations. The American College of Radiology (ACR) stated: “A study into reports of extravasation found that <0.001% of diagnostic nuclear medicine extravasations result in temporary symptoms of any kind.”^1^ One member stated: “Gamma ray emitting radiotracer which has very negligible effects on the skin if infiltrated and is not at risk of being harmful.” Further, the ACR compared contrast media and chemotherapeutic administrations to nuclear medicine administrations as follows when stating: “They are not analogous to administrations of radiopharmaceuticals, which are typically small volume, without inherent properties harmful to tissue.” In discussing diagnostic extravasations, the ACR also stated: “it would be unlikely if not impossible to meet the §35.3045(b) standard with typical diagnostic nuclear medicine agents.” Leading members of the Society of Nuclear Medicine and Molecular Imaging (SNMMI) when referencing diagnostic extravasations stated “An extravasated radiopharmaceutical administration would never deliver 0.5 Sv (50 rem) to any whole organ or tissue, including the skin.”^2^ And the Health Physics Society stated: “there is no evidence that infiltration of radiopharmaceuticals carries any health consequences for the patient or the general public” and “designating extravasations as a Medical Event would call for a dose estimation, which is far from a trivial process. Use of simplified techniques do not effectively account for removal of the pharmaceutical from the injection site and the time varying geometry of the source, though they can be modified to do so with additional data collection.”^3^ These positions reveal commonly held hypotheses regarding diagnostic extravasations—they do not cause patient harm, their resulting absorbed dose cannot exceed regulatory reporting limits and threshold limits that can lead to adverse tissue reactions, and dosimetric characterization is beyond the capabilities of most nuclear medicine centers.

We analyzed the three hypotheses considering data from: (1) Current literature, (2) Databases of adverse events and vigilance reports of patient harm, and (3) Five patient cases of significant extravasations in our centers for whom dosimetry calculations were performed. Our findings suggest new hypotheses related to the impact of nuclear medicine extravasations. Diagnostic radiopharmaceutical extravasations can result in patient harm, can result in absorbed doses that exceed current regulatory reporting limits and threshold limits that can lead to adverse tissue reactions, and appropriate dosimetry methods can be applied with minimal resources to provide effective characterization of extravasations across most centers.

## Methods

### Literature Review

We conducted a review of the medical literature looking for evidence of patient consequences from diagnostic extravasations. We used search word(s) such as: radiopharmaceutical administration, infiltration, extravasation, paravenous injections, interstitial injections, and dose tissuing. We then searched for evidence of harm or potential for harm as a result of diagnostic extravasations. Harm or potential harm included: unnecessary additional exposure to radiation through repeat imaging, delays in treatment, false positives or negatives that led to additional patient intervention, incorrect diagnosis, or incorrect treatment assessment, and deterministic effects or adverse tissue reactions.

### Database Search Strategy

We conducted a systematic search of the Food and Drug Administration's (FDA) Adverse Event Reporting System (FAERS) (4) Public Dashboard for U.S. adverse events and the EudraVigilance (EV) (5) database for adverse drug reactions in the European Economic Area (EEA) to find relevant reports of diagnostic nuclear medicine extravasations through November 2019.

FAERS is a compilation of medication error reports voluntarily submitted to FDA by healthcare professionals, consumers, and manufacturers. The FAERS search included adverse drug reaction reports from 1968 to 2019, based on FDA-approved radiopharmaceuticals (6) or trade names. The search methodology begins with “Search.” Select “Search by Product” and insert FDA-approved radiopharmaceutical or trade name. Initiate search. Select “listing of cases.” Results were filtered by selecting “reaction group.” These results were further filtered by selecting the group: “General Disorders and Administration Site Conditions.” Within this group, additional filtering was employed by selecting “Reaction” and then expanding the “General Disorders and Administration Site Conditions.” In “Number of Cases” the results were further filtered by selecting the terms: “extravasation,” “administration site extravasation,” “application site extravasation,” “catheter site extravasation,” “infusion site extravasation,” “injection site extravasation,” “medical device site extravasation,” and “tissue infiltration.” Results were manually exported, duplicates were removed, and findings summarized.

EV is the pharmacovigilance database to manage the collection and analysis of suspected adverse drug reactions authorized within the EEA. Reports are submitted electronically by national regulatory authorities and by pharmaceutical companies that hold licenses for medicines. The search methodology begins with “Search.” Select “Suspected adverse drug reaction reports for Products” and select a letter or number from which to browse a radiopharmaceutical name or trade name. Initiate the search by selecting the name. Select the “Line Listing” tab to access the filtering conditions for individual cases identified in EV for the radiopharmaceutical. Within filtering conditions, from the “Reaction Groups” dropdown list, select “General disorders and administration site conditions. From the “Reported Suspected Reaction” dropdown list, select terms “extravasation,”, “administration site extravasation,” “application site extravasation,” “catheter site extravasation,” “infusion site extravasation,” “injection site extravasation,” “medical device site extravasation,” and “tissue infiltration.” Using the “Gateway Date” dropdown list, select a year and select “Run Line Listing Report.” If individual case reports were input into the database for the given year, reports will be listed. This search process was repeated for each year from 1994 to 2019 for each radiopharmaceutical. Results were manually exported, duplicates were removed, and findings were summarized.

### Patient Management Effects

We also analyzed data from two nuclear medicine centers – Carilion Clinic, Roanoke, VA and the University of Tennessee Medical Center, Knoxville, TN. In both centers, injection quality is routinely monitored by technologists using bilateral, external radiation detectors (Lara^®^ System, Lucerno Dynamics, Cary, NC, USA) that provide a real time display of counts after injection and prior to imaging. Once the administration is complete, nuclear medicine physicians also review this information now in the form of a time-activity curve (TAC) to evaluate the quality of radiopharmaceutical administration. Repeat imaging in these centers is routinely ordered for patients with extravasations that the interpreting nuclear medicine physician suspects would negatively affect the diagnostic value of the imaging study. For this report we selected five representative cases involving extravasated diagnostic radiopharmaceuticals: ^18^F-Fluorodeoxyglucose (^18^F-FDG) for Positron Emission Tomography/Computed Tomography (PET/CT) (*N* = 4) and ^99m^Tc-Methylene diphosphonate (^99m^Tc-MDP) for Single Photon Emission Computed Tomography (SPECT) (*N* = 1). Acquisition and reconstruction parameters for each site are described below.

At Carilion Clinic, when performing PET/CT the standard injected activity was 10mCi 18F-FDG using a standard step and shoot acquisition routine with axial range matched to the CT FOV. For CT Acquisition, data were acquired using 120 kVp with CareDose 4D. Data were acquired using a 20 × 0.6 mm detector setting with 5.0 mm slice thickness. For Bone SPECT the standard injected activity was 25mCi 99mTc-MDP using a LEHR collimator and matrix size of 256 × 256 with a zoom of 1. SPECT data were acquired using 30 views in a non-circular (body contour) orbit with an acquisition time of ~20 s per view. For CT Acquisition, data were acquired using 130 kVp with CareDose 4D and pitch of 1.5 and detector settings of 16 × 1.2 mm with 3 mm slice thickness. For visualization, both B80s AC for ECT and B50s medium sharp with bone window kernels were employed.

At University of Tennessee Medical Center, when performing PET/CT the standard injected activity was 10 mCi of 18F-FDG ±20%. Data were acquired using continuous bed motion acquisition with a bed speed of 1.5 mm/s. Reconstruction was performed using point spread function resolution recovery with time of flight (UltraHD PET, Siemens Healthineers, Malvern, PA). A matrix size of 200 × 200 was used with 3 iterations and 21 subsets and a Gaussian filter of 5 mm FWHM was applied. For CT, data were acquired using 120 kVp with CareDose 4D using a 5 mm acquisition technique reconstructed to 4 mm slice thickness and using a standard abdomen kernel.

All patients consented to publication of their images or were granted a waiver of consent by the local institutional review board. Attempts were made during the repeat studies to ensure that imaging parameters and patient preparation were as consistent as possible with the extravasated procedure to help assess extravasation effects. PET/CT scans were performed on the Biograph mCT camera (Siemens Healthineers, Knoxville, TN). SPECT scans were performed on the Symbia Intevo Bold™ camera (Siemens Healthineers, Knoxville, TN). Extravasated and repeated images were interpreted on the day of the procedure by a trained nuclear medicine physician and findings from the scan were recorded in the standard department reporting system.

### Tissue Dosimetry

We calculated absorbed tissue dose using methods previously described in the literature (7). When considering extravasations, the calculated dose is for that tissue which contains residual radiopharmaceutical, and the absorbed energy comes from the radiopharmaceutical itself—self dose. As the radiopharmaceutical decays, it releases energy in the form of photons, x-rays, electrons, positrons, etc., with specific energy deposition characteristics dependent on the particular isotope. Furthermore, these primary emissions lead to secondary emissions through excitation of the surrounding tissue. Unlike the emissions used for imaging (i.e., 511 keV gammas for PET), many of these emissions will not penetrate the local tissue; their energy may be absorbed completely within a short distance.

To calculate self-dose to tissue from extravasation, three pieces of information are needed: absorbed energy per radioactive decay, initial source activity, and the rate at which the activity is decreasing due to the combination of physical half-life and biological clearance.

Using the methods described by Osborne et al. (7), we used the GATE Monte-Carlo framework to simulate absorbed energy per decay for both 18F and 99mTc. Simulations consisted of 5-gram spheres of water uniformly containing 1 MBq of each isotope as an ion source. Spheres with a mass of 5 g were chosen to strike a balance between accuracy of localized dosimetry vs. very high absorbed doses in very small tissue volumes. Absorbed energy within the sphere was then converted to absorbed dose rate in Gy/MBq-min.

Effective half-life is the combination of the isotope-specific physical half-life and the rate of biological clearance. This combination can be found using multiple image-based measurements of the area (8–13), or through measurements with a scintillation counter or other radiation detection system (14). We used data from external radiation detectors to estimate the effective half-life as described previously by Osborne et al. (7). The external detector data indicates the rate of change of localized radioactivity over time, to which we fit a mono-exponential function to obtain the effective half-life. The initial activity within the extravasation can be estimated by extrapolating a quantitative measurement of the injection site backwards to time zero using the effective half-life. We used quantitative PET imaging or qualitative image assessments for the activity estimate at imaging time and extrapolated to time zero using the effective half-life.

With the above values determined, calculation of absorbed dose is straight forward (Equations 1 and 2). Total absorbed energy in Joules is found by the multiplication of the initial activity in Bq, the absorbed energy per decay in Joules (1 keV = 1.602 × 10^−16^ J), the effective half-life in seconds, and one divided by the natural logarithm of two. Total absorbed dose is then the ratio of total absorbed energy divided by tissue mass in kilograms.

(1) $$\begin{array}{r} Total\;Absorbed\;Energy\;\left(J\right)=\;\frac{A_{0}\;\times \;Q\;\times \;T_{\frac{1}{2},eff}}{\ln\;2}, \end{array}$$

where A_0_ is the initial activity in Bq, Q is the absorbed energy per decay in J, and T_1/2, eff_ is the effective half-life in sec.

(2) $$\begin{array}{r} Total\;Absorbed\;Dose\;\left(Gy\right)=\;\frac{E}{M}, \end{array}$$

where E is the total absorbed energy in J and M is the tissue mass in kg.

For skin, we used the computer code VARSKIN 6 (15) to calculate the shallow dose equivalent to 10 cm^2^ of skin overlaying the extravasation.

## Results

### Literature Review

We found 58 peer-reviewed articles that show how diagnostic extravasation can harm or have harmed patients. Extravasations have a negative effect on the sensitivity of PET/CT. The clinical implications of an extravasation on a PET/CT study for the management of cancer patients include under staging the disease (16–23), over staging the disease (16–18, 20, 24–45), therapeutic procedure planning errors (46), and therapy assessment errors (22, 45, 47–57). Extravasations have negative patient management implications also in PET/CT procedures that are used for indications other than oncology. For example, an extravasation that occurred during a myocardial perfusion study can lead to either a false positive or false negative misinterpretation with serious consequence for patient management (19, 58–61); an extravasation during an FDG neurological function study would adversely affect the reported results by limiting the FDG uptake in the brain (62); an extravasation during amyloid plaque imaging for Alzheimer's disease and dementia diagnosis can cause poor image quality (63) and during fever of unknown origin study may compromise imaging sensitivity and diagnostic capability.

Extravasations of diagnostic radiopharmaceuticals during gamma camera procedures have similar implications to those found in extravasated PET/CT procedures: misinterpretation of results may lead to patient harm, unnecessary invasive procedures, and additional exposure to radiation from repeat scans. In a renal scan/glomerular filtration rate studies extravasations can cause false-positive findings, can require repeat procedures (32), and may not be visible in the imaging FOV (64, 65). In Tc-99m Sestamibi studies an extravasated injection can result in a scan with poor-quality images, mask ischemia, and can lead to an inappropriate investigation for malignancy (58). In multigated acquisition (MUGA) studies used to assess the impact of a patient's chemotherapy treatment on myocardial function, an extravasation can result in suboptimal radiolabeling of blood cells with corresponding increased amounts of residual, unreacted free pertechnetate (66) and lead to inappropriate cessation of chemotherapy treatment. In dopamine transporter imaging studies assessing Parkinson's disease an extravasation of Ioflupane I-123 can confound the dopamine transporter study results (67, 68). In Ventilation Perfusion (V/Q) studies an extravasation creates the opportunity for false negative interpretations (69) with potential serious patient implications (70, 71). In planar bone scanning an injection issue can lead to misinterpreting an extravasation for pathologic findings, false positive lymph node uptake, and “Compton scatter” possibly resulting in misinterpretation of significant breast abnormality (72). Additionally, patients can experience adverse tissue reactions from significant diagnostic extravasations (20, 39, 73).

### Database Search

Examining the FAERS database, we found 21 adverse events which included diagnostic radiopharmaceutical extravasation as a factor, despite the NRC's reporting exemption in the United States. Table 1 contains a listing of these cases.

**Table 1:** FAERS listing of cases.

**Radiotracer**	**Reaction(s)/event(s)**	**Serious/seriousness**	**Outcomes**
Lymphoseek (Tc-99m Tilmanocept)	Injection site hemorrhage, injection site pain, incorrect dose administered	Non-serious	Non-serious
Myoview (Tc-99m tetrofosmin)	Extravasation	Non-serious	Non-serious
Myoview (Tc-99m tetrofosmin)	Extravasation	Non-serious	Non-serious
Technescan Hdp (oxidronate)	Injection site erythema, injection site extravasation, pyrexia, toxicity to various agents, chills, injection site inflammation, injection site edema, cellulitis	Serious	Required intervention
Technescan Hdp (oxidronate)	Extravasation, skin reaction	Serious	Other outcomes
Technescan Hdp (oxidronate)	Extravasation, injection site swelling, injection site erythema	Serious	Required intervention
Technescan Hdp (oxidronate)	Cellulitis, product administration error, injection site swelling, injection site extravasation, injection site erythema, injection site pain	Serious	Hospitalized
Technescan Hdp (oxidronate)	Injection site cellulitis, injection site extravasation, product administration error	Serious	Other outcomes
Technescan Hdp (oxidronate)	Product administration error, phlebitis, extravasation	Serious	Other outcomes
Technescan MAG3 (mertiatide)	Extravasation	Non-serious	Non-serious
Technescan MAG3 (mertiatide)	Injection site extravasation, product administration error	Non-serious	Non-serious
Technescan MAG3 (mertiatide)	Pain, injection site swelling, injection site pain, product administration error, swelling, injection site extravasation	Non-serious	Non-serious
Technetium Tc-99m Etidronate	Peripheral edema, extravasation	Non-serious	Non-serious
Technetium TC-99m Medronate (MDP)	Hypoaesthesia, paraesthesia, peripheral swelling, injection site extravasation, pain in extremity	Serious	Other outcomes
Technetium Tc-99m Sestamibi (Cardiolite)	Injection site extravasation	Non-serious	Non-serious
Technetium Tc-99m Sestamibi (Cardiolite)	Infusion site extravasation, infusion site swelling	Non-serious	Non-serious
Technetium Tc-99m Sestamibi (Cardiolite)	Product administration error	Non-serious	Non-serious
Technetium Tc-99m Sestamibi (Cardiolite)	Product administration error	Non-serious	Non-serious
Technetium Tc-99m Sestamibi (Cardiolite)	Retching, feeling hot, vomiting, nausea, loss of consciousness, seizure, cardiac arrest, infusion site extravasation, presyncope, abdominal pain, electrocardiogram St segment depression	Serious	Other outcomes, life threatening
Technetium Tc-99m Sestamibi (Cardiolite)	Pain, injection site extravasation, ecchymosis	Non-serious	Non-serious
Technetium Tc-99m Sestamibi (Cardiolite)	Discomfort, necrosis, peripheral nerve injury, injection site extravasation	Serious	Other outcomes

Examining the EV database, we found 17 diagnostic radiopharmaceutical extravasation safety reports in Europe as seen in Table 2. For the EEA, seriousness includes: “death,” “life-threatening,” “requires hospitalization/prolongation of hospitalization,” “results in disability/incapacity,” “congenital anomaly/birth defect,” and “other.”

**Table 2:** EV search line listing report.

**Radiotracer**	**Reaction(s)/event(s)**	**Serious/seriousness**	**Outcomes**
DaTscan (I-123)	Product administration error, no adverse event	Serious	Other Outcomes
Fluorodeoxyglucose F-18	Extravasation, peripheral swelling	Non-serious	Recovered/resolved
Fluorodeoxyglucose F-18	Extravasation, injection site pain	Non-serious	Recovered/resolved
Fluorodeoxyglucose F-18	Extravasation, hypoaesthesia, injection site erythema	Non-serious	Recovered/resolved
Fluorodeoxyglucose F-18	Application site extravasation	Non-serious	Unknown
Fluorodeoxyglucose F-18	Injection site extravasation, injection site pain	Non-serious	Recovered/resolved
Myoview (Tc-99m tetrofosmin)	extravasation	Serious	Other Outcomes
Technescan Hdp (oxidronate)	Product administration error, injection site extravasation, injection site inflammation	Serious	Other Outcomes
Technetium 99m oxidronate	Extravasation	Unknown	Unknown
Technetium 99m oxidronate	Extravasation	Other	Recovered/resolved
Technetium 99m oxidronate	Cold sweat, injection site extravasation, pain	Unknown	Recovered/resolved
Technetium 99m oxidronate	Injection site extravasation	Unknown	Recovered/resolved
Technetium 99m oxidronate	Injection site extravasation, injection site ulcer	Other	Unknown
Technetium 99m oxidronate	Erysipelas, injection site extravasation, injection site inflammation	Caused or prolonged hospitalization	Recovered/resolved
Technetium 99m oxidronate	Injection site extravasation, edema, rash erythematous	Disability	Recovered/resolved
Technetium TC-99m Pentetate	Injection site extravasation, injection site edema, injection site pain	Other	Recovered/resolved
Technetium Tc-99m Sestamibi	Extravasation, swelling	Unknown	Unknown

### Examples of Patient Management Effects

Between April 1 and September 30, 2019, Carilion Clinic enrolled 1,023 patients. We identified 34 cases of extravasation (3.3%). Of these, 11 were assessed to be moderate to significant based on TAC data and visual assessment of the images. We estimated that all 11 of these patients had more than 5% of their injected activity extravasated. Three of the 11 patients were asked to provide consent to publish their extravasated and non-extravasated images and results.

For University of Tennessee Medical Center (UTMC) patients, we performed a retrospective analysis of more than 3,534 patients with monitored injections under an exempt protocol that was reviewed and approved by the University of Tennessee Graduate School of Medicine Institutional Review Board (IRB # 4607). Of those reviewed, 100 imaging studies (2.8%) were determined to have residual radioactivity at the site of injection. Of those, thirteen were moderate to significant based on TAC information and visual image assessment. Two cases were selected for inclusion in this work.

### Patient A

**Figure 1:**
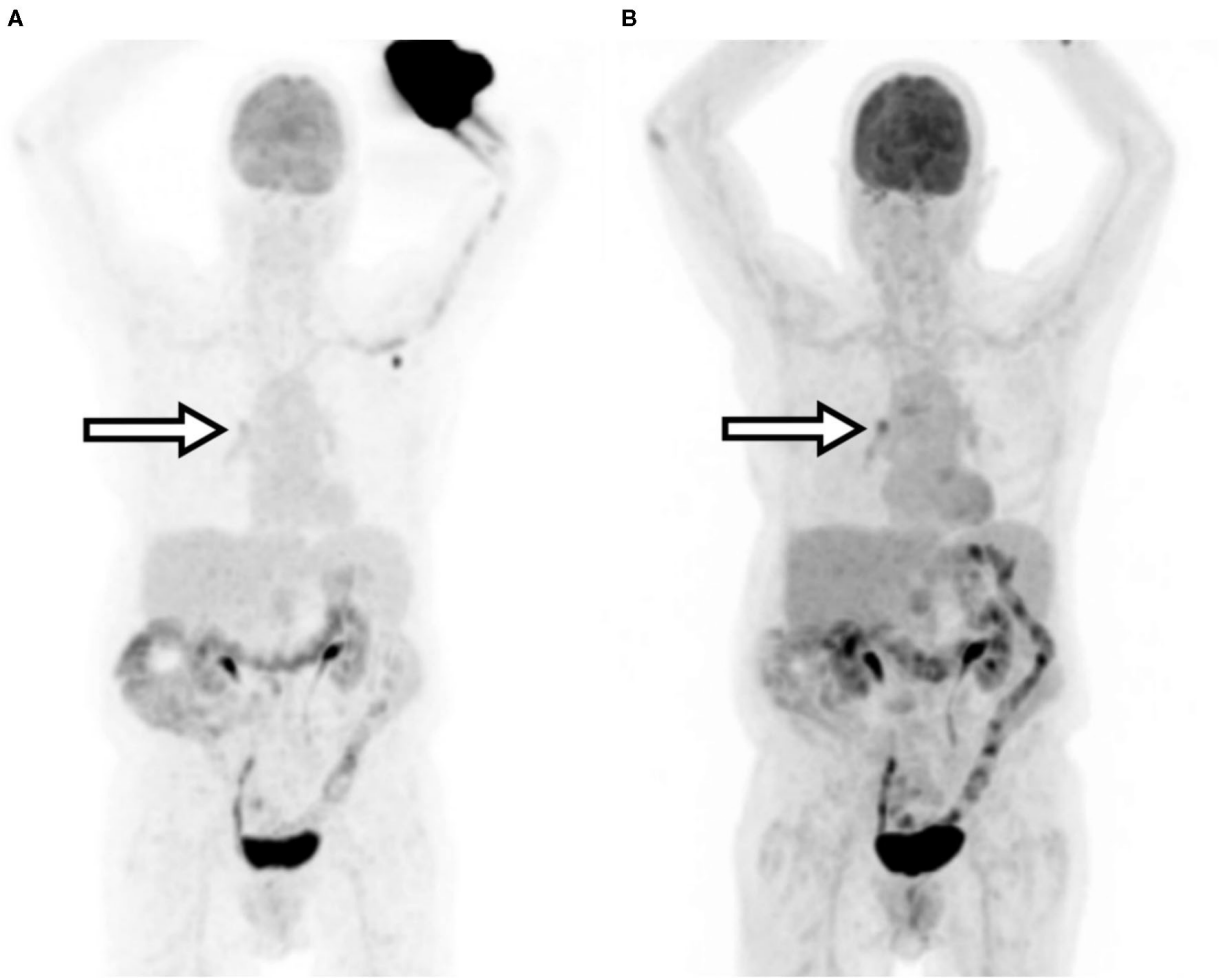
Patient A maximum intensity projection (MIP) images. **(A)** Extravasated injection. **(B)** Repeated imaging. Arrows indicate the clinically relevant area of uptake.

A 79-year-old female presented with stage 2B T3N1M0 pancreatic cancer. The tumor arose in the pancreatic head but was felt to be unresectable due to neurovascular involvement. During an FDG-PET/CT scan for staging, the patient experienced a significant radiopharmaceutical extravasation and was reimaged the following day. The patient subsequently received chemoradiation since she was not considered a surgical candidate for tumor resection. Procedure parameters and images are compared in Table 3 and Figure 1.

**Table 3:** Procedure parameters for Patient A.

**Procedure parameter**	**Staging PET/CT Extravasated injection**	**Repeat imaging Ideal injection**
Day	0	1
Hours of fasting	>8	>8
Strenuous activity 24 h prior	No	No
Cold exposure 24 h prior	No	No
Concomitant medication	None	None
Blood glucose level	92 mg/dL	143 mg/dL
Weight	72.6 kg	72.6 kg
Injection start time	7:12 a.m.	6:42 a.m.
Net delivered FDG dose	378 MBq	378 MBq
Flush volume	30 mL	20 mL
PET/CT bed protocol	Flowmotion at 1.6 cm/min	Flowmotion at 1.6 cm/min
Imaging start time	8:20 a.m.	7:40 a.m.
Uptake time	68 min	58 min
Physician assessment of injection quality	Significant extravasation	Ideal injection
Clinical finding	Axillary nodal uptake	No nodal uptake
Pancreatic Tumor SUV_max_	1.86	4.56

### Patient B

**Figure 2:**
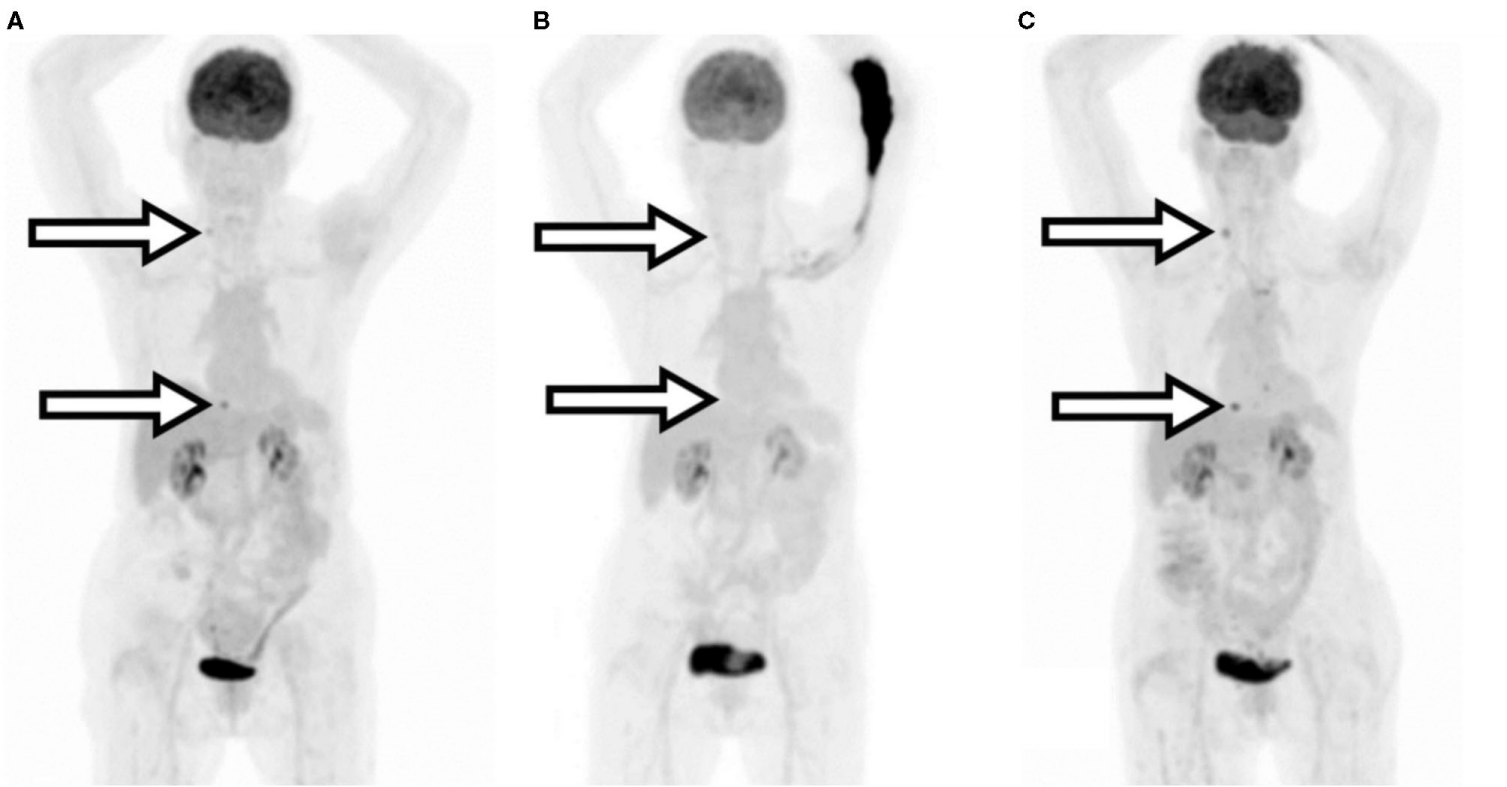
Patient B MIP images. **(A)** Initial re-staging imaging. **(B)** Second re-staging extravasated injection. **(C)** Repeated imaging. Arrows indicate the areas of relevant uptake which were not visible after extravasation.

A 69-year-old female with a history of a T2N2aM0 breast cancer who subsequently developed metastatic skeletal disease. The patient received their first re-staging ^18^F-FDG PET/CT and was noted to have oligometastatic skeletal metastases. The patient received radiotherapy and then a second re-staging FDG PET/CT 15 weeks later. This second re-staging study was compromised by a significant radiopharmaceutical extravasation and was repeated 3 days later. Procedure parameters and images are compared in Table 4 and Figure 2. The extravasated image SUV_max_ values were understated by 53–73%.

**Table 4:** Procedure parameters for Patient B.

**Parameters**	**Initial re-staging PET/CT Ideal injection**	**Follow-up restaging PET/CT Extravasated injection**	**Repeated imaging Ideal injection**
Day	−103	0	3
Hours of fasting	Unknown	>8	>8
Strenuous activity 24 h prior	Unknown	None	None
Cold exposure 24 h prior	Unknown	None	None
Concomitant medication	Unknown	None	None
Blood glucose level	121 mg/dL	100 mg/dL	108 mg/dL
Weight	53.5 kg	51.7 kg	51.7 kg
Injection start time	1:11 p.m.	2:50 p.m.	7:31 a.m.
Net delivered FDG dose	359 MBq	370 MBq	343 MBq
Flush volume	45 mL	20 mL	20 mL
PET/CT bed protocol	Flowmotion at 1.6 cm/min	Flowmotion at 1.6 cm/min	Flowmotion at 1.6 cm/min
Imaging start time	2:08 p.m.	3:52 p.m.	8:52 a.m.
Uptake time	57 min	62 min	81 min
Physician assessment of injection quality	Ideal injection	Significant extravasation	Ideal injection
Lesion 1 SUV_max_	5.75	1.68	6.30
Lesion 2 SUV_max_	2.81	1.10	2.36

### Patient C

**Figure 3:**
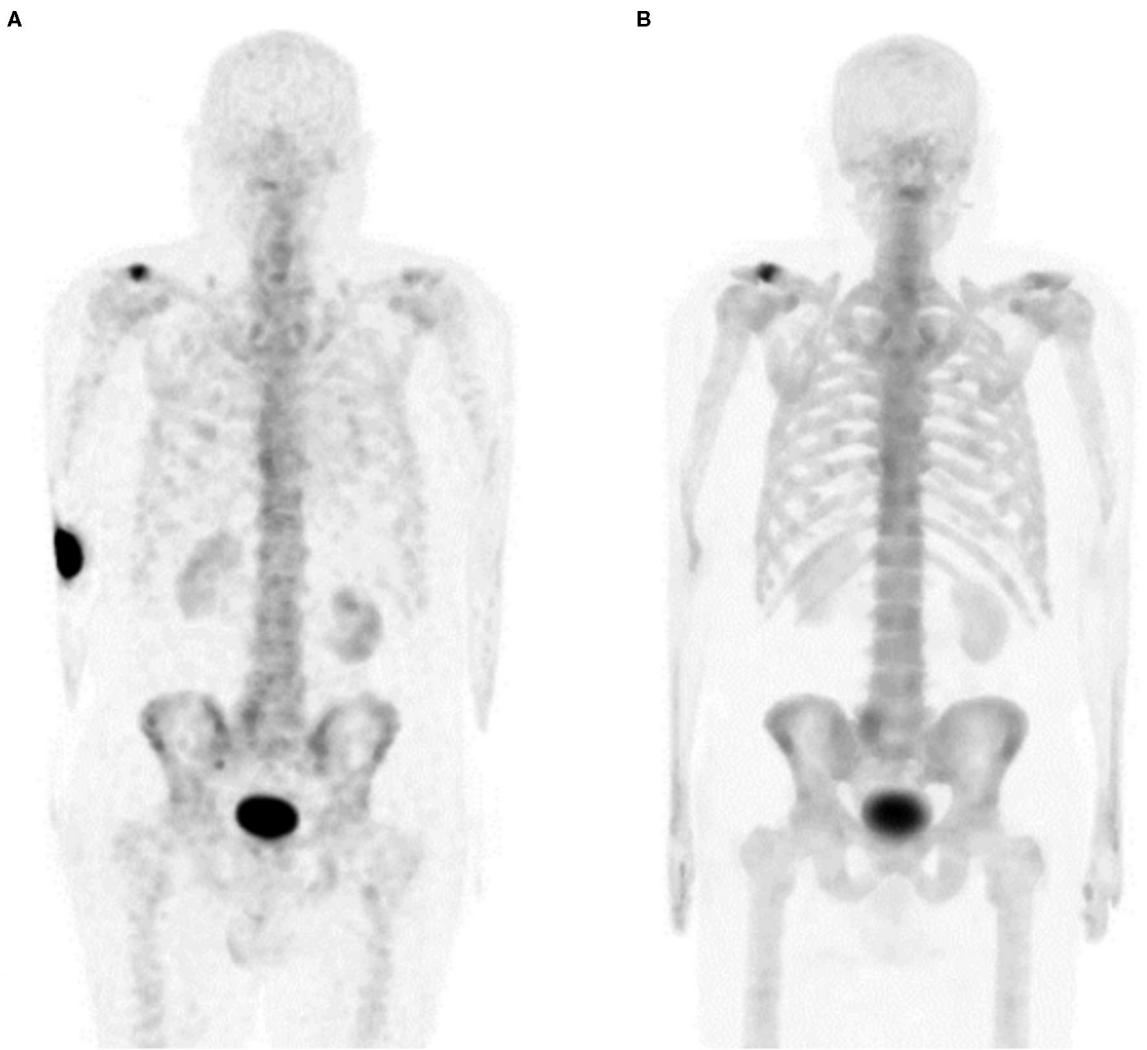
Patient C MIP images. **(A)** Extravasated injection. **(B)** Repeated imaging.

An 81-year-old male presented with a history of clinical stage T1cN0M0 prostate cancer. Post-prostatectomy, the patient had persistently elevated prostate-specific antigen levels and a ^99m^Tc-MDP bone scan was ordered to look for metastatic disease. The bone scan image was deemed non-diagnostic by the radiologist due to significant extravasation and was not interpreted. The study was repeated 2 days later. Benign degenerative disease was noted, but no metastatic bone lesions were found. Procedure parameters and images are compared in Table 5 and Figure 3.

**Table 5:** Procedure parameters for Patient C.

**Parameters**	**Initial bone scan Extravasated injection**	**Repeated imaging Ideal injection**
Day	0	2
Concomitant medication	None	None
Weight	95.3 kg	95.3 kg
Injection start time	7:35 a.m.	8:45 a.m.
Net delivered MDP dose	969 MBq	1.01 GBq
Flush volume	0 mL	0 mL
Imaging start time	10:59 a.m.	12:24 p.m.
Uptake time	3 h 24 min	3 h 39 min
Physician assessment of injection quality	Significant extravasation	Ideal injection
Clinical finding	Non-diagnostic image	No evidence of skeletal disease

### Patient D

**Figure 4:**
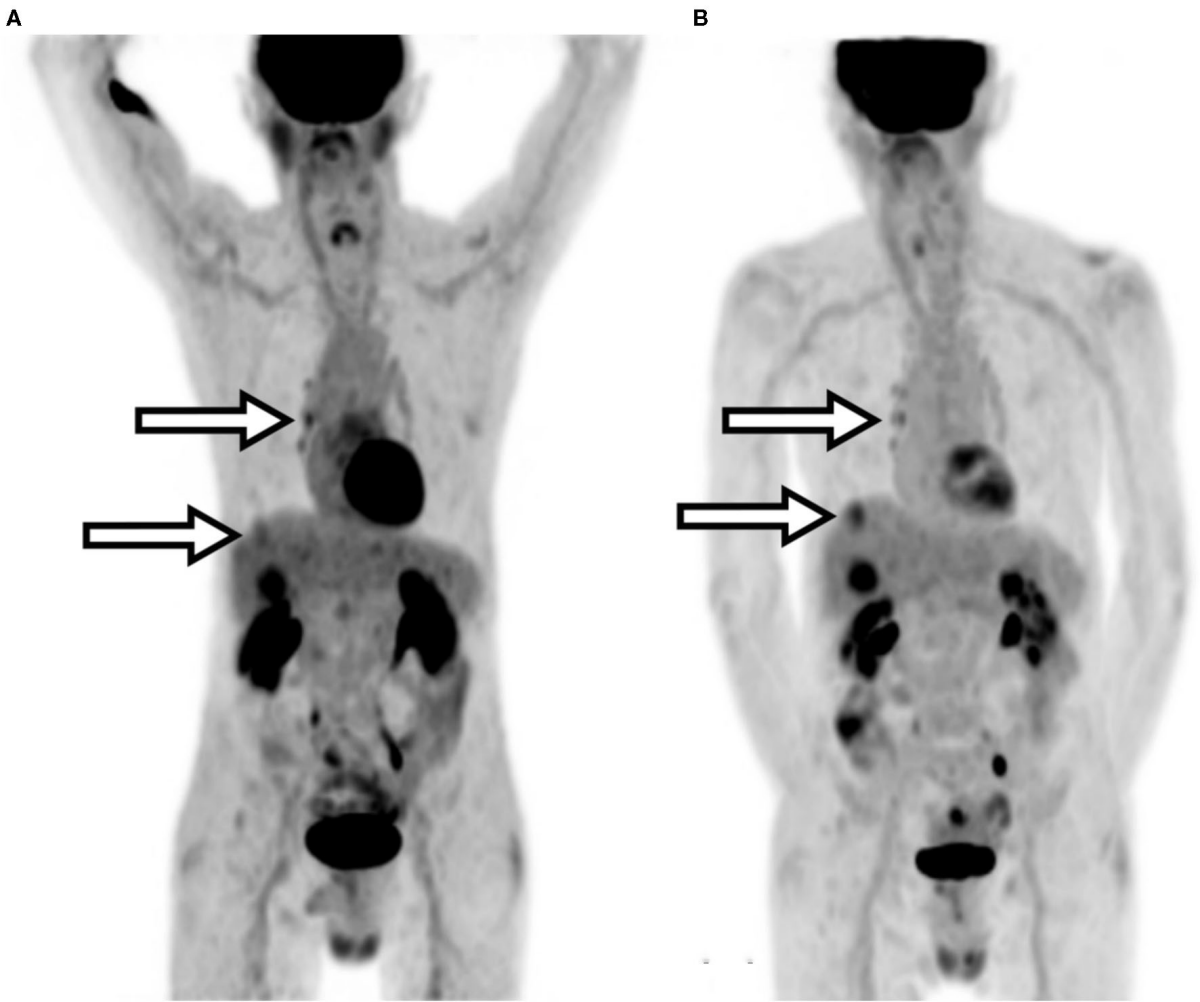
Patient D MIP images. **(A)** Extravasated injection. **(B)** Repeated imaging. Arrows indicate the areas of clinically relevant increased uptake.

An 80-year-old male presented with a history of bladder carcinoma that had metastasized to the liver. Initial follow-up imaging was determined to be non-diagnostic due to a significant extravasation observed on imaging and TAC data. Repeat ^18^F-FDG PET/CT imaging of the patient was performed the following day. Repeat imaging confirmed disease progression and identified additional uptake not seen in the prior extravasated scan—including an upper liver lesion, increased hilar node activity, and prostate uptake. Quantitative results showed an average increase in SUV_max_ calculations of ~25%. Procedure parameters and images are compared in Table 6 and Figure 4.

**Table 6:** Procedure parameters for Patient D.

**Parameters**	**Restaging PET/CT Extravasated injection**	**Repeat imaging Ideal injection**
Day	0	1
Hours of fasting	>8	>8
Strenuous activity 24 h prior	None	None
Cold exposure 24 h prior	None	None
Concomitant medication	None	None
Blood glucose level	96 mg/dL	113 mg/dL
Weight	57.6 kg	57.6 kg
Injection start time	1:54 p.m.	12:56 p.m.
Net delivered FDG dose	362.6 MBq	343 MBq
Flush volume	10 mL	20 mL
PET/CT bed protocol	Flowmotion at 2 cm/min	Flowmotion at 2 cm/min
Imaging start time	3:05 p.m.	2:19 p.m.
Uptake time	71 min	83 min
Physician assessment of injection quality	Significant extravasation	Ideal injection
Lesion 1 SUV_max_	5.7	7
Lesion 2 SUV_max_	2.8	5

### Patient E

**Figure 5:**
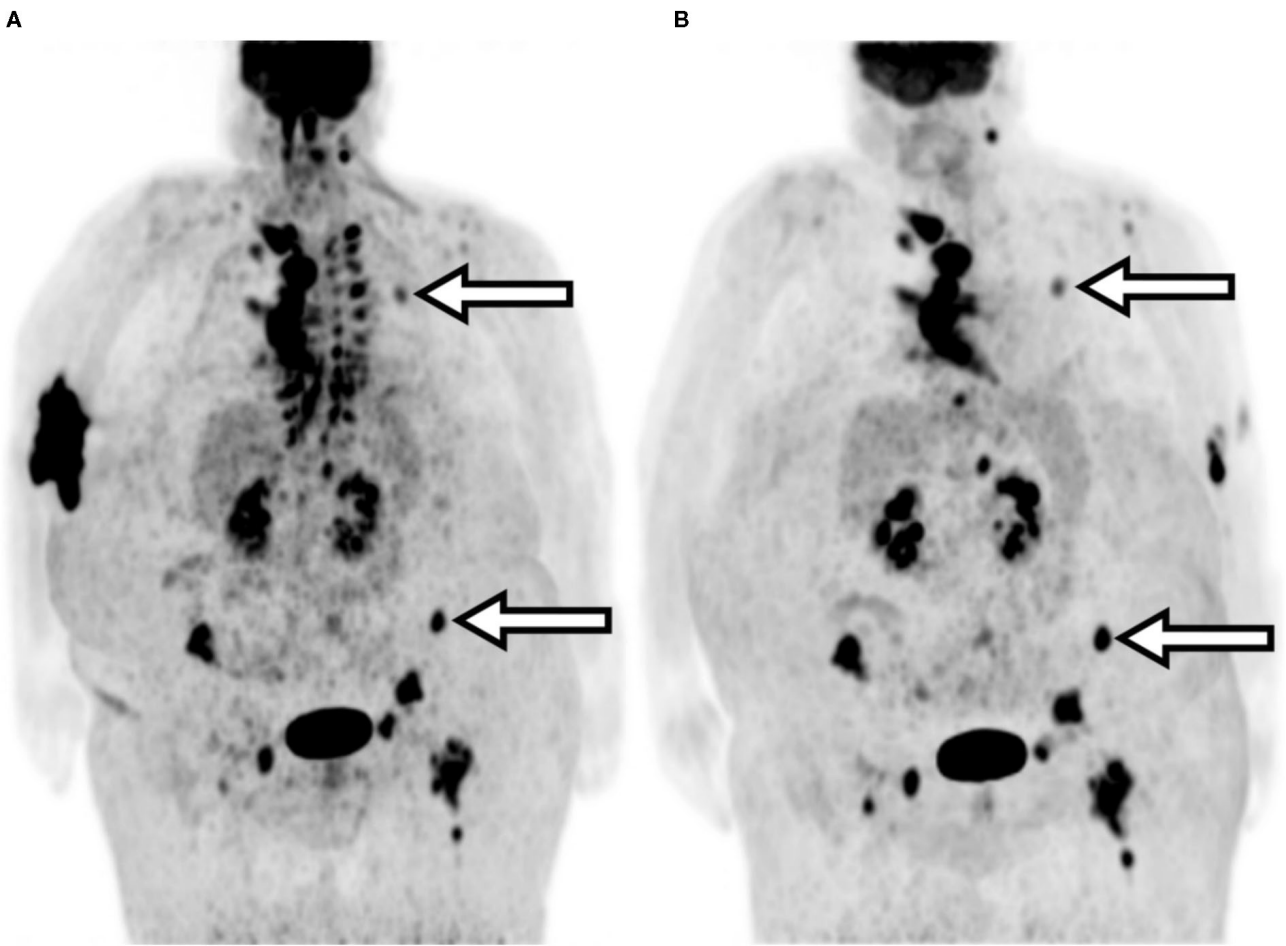
Patient E MIP images. **(A)** Extravasated injection. **(B)** Repeated imaging. Arrows indicate areas of relevant uptake between the two scans.

A 61-year-old female presented with a history of breast cancer and malignant right pleural effusion. Follow-up imaging identified possible bone involvement and additional ^18^F-FDG PET/CT imaging was ordered. The restaging PET/CT images were deemed non-diagnostic due to a significant extravasation. Repeat PET/CT imaging was ordered, and the patient was imaged 5 days later. Repeat imaging showed evidence of a minor extravasation which was supported by TAC data but indicated diffuse metastatic disease with bone involvement—confirming further disease progression. Additionally, the repeated imaging clearly identified a left adrenal lesion that had appeared equivocal on the previous extravasated image and increased SUV values. Procedure parameters and images are compared in Table 7 and Figure 5.

**Table 7:** Procedure parameters for Patient E.

**Parameters**	**Restaging PET/CT Extravasated injection**	**Repeat imaging Ideal injection**
Day	0	5
Hours of fasting	>8	>8
Strenuous activity 24 h prior	None	None
Cold exposure 24 h prior	None	None
Concomitant medication	None	None
Blood glucose level	90 mg/dL	82 mg/dL
Weight	117.9 kg	117.9 kg
Injection start time	1:58 p.m.	11:59 a.m.
Net delivered FDG dose	373.7 MBq	392.2 MBq
Flush volume	40 mL	20 mL
PET/CT bed protocol	Flowmotion at 1.5 cm/min	Flowmotion at 1.5 cm/min
Imaging start time	3:14 p.m.	1:13 p.m.
Uptake time	76 min	74 min
Physician assessment of injection quality	Significant extravasation	Ideal injection
Lesion 1 SUV_max_	9.1	11.2
Lesion 2 SUV_max_	3.5	4.2

### Tissue Dose

Dosimetry results for the five extravasations are shown in Table 8. Skin and tissue effects were not observed post-imaging procedure, as expected due to the latent effects of ionizing radiation on skin and tissue.

**Table 8:** Dosimetry results.

	**Patient A**	**Patient B**	**Patient C**	**Patient D**	**Patient E**
Isotope	^18^F	^18^F	^99m^Tc	^18^F	^18^F
Administered activity (MBq)	378	370	969	363	374
Average energy per decay (keV)	240	240	18	240	240
Average energy per decay (J)	3.85 ×10^−14^	3.85 ×10^−14^	2.88 ×10^−15^	3.85 ×10^−14^	3.85 ×10^−14^
Initial extravasation activity (Bq)	378 ×10^6^	285 ×10^6^	485 ×10^6^	98 ×10^6^	374 ×10^6^
Effective half-life (sec)	2,262	1,326	21,624	1,074	1,344
Total decays	1.23 ×10^12^	5.45 ×10^11^	1.51 ×10^13^	1.52 ×10^11^	7.25 ×10^11^
Total absorbed energy (keV)	2.96 ×10^14^	1.31 ×10^14^	2.57 ×10^14^	3.64 ×10^13^	1.74 ×10^14^
Total absorbed energy (J)	0.047	0.021	0.043	0.006	0.028
Absorbed dose to 5 g of tissue (Gy)	9.4	4.2	8.6	1.2	5.6
Shallow dose equivalent to 10 cm^2^ of skin (Sv)	4.2	1.9	<0.1	0.5	2.5

## Discussion

### Reasons for the Commonly Held Hypotheses

Several potential reasons could explain these commonly held hypotheses regarding diagnostic extravasations. In the United States, there are no mandatory requirements to report extravasations. Furthermore, the primary accreditation bodies do not audit the frequency of extravasations. As a result, centers do not routinely follow extravasated patient, nor do they routinely perform dosimetry on extravasated patients. This hesitancy to perform dosimetry may not be exclusive to the United States. In a comprehensive literature review of the consequences of extravasations, van der Pol et al.found that in 3,016 reported cases of diagnostic extravasations, only three cases reported dosimetry and patient follow-up. All three cases resulted in adverse tissue reactions. The authors stated that “lack of clinical follow-up after diagnostic nuclear medicine scans, but also conservative attitude toward reporting and publishing of complications may have possible lead *[sic]* to under-reporting of skin lesions” (20). Submitted public comments to the NRC also suggest a lack of awareness of the non-penetrating energy emissions associated with routinely used isotopes like ^99m^Tc and ^18^F. It is often overlooked in standard nuclear medicine practice, that conversion electrons, Auger electrons, low-energy photons, and positrons that result in a minimal dose to tissue when properly administered and circulating in the entire body, can result in extremely high absorbed doses when localized during an extravasation. It also may be easy to summarily dismiss the possibility of localized damage, given the standard assumptions based on whole-body exposures during diagnostic procedures involving ideal radiopharmaceutical administrations. However, low-energy and low-intensity emissions must be considered when calculating dose due to extravasation sites, even if they are routinely ignored in the context of diagnostic nuclear imaging. Many comments note no evidence of harm to patients immediately after an extravasation. These comments indicated a lack of awareness of the typical time required for radiation injury to manifest in patient skin and tissue. Skin reactions can take days and often the skin may not be affected, even when the tissue dose is high. And tissue reactions can take months or years and would not be easily attributed to extravasations, especially when the patient and their treating physician may not be experienced in radiation protection principles.

ACMUI statements are another important reason why these commonly held hypotheses exist. Over 300 public comments regarding the extravasation petition to the NRC refer to the published ACMUI positions. These positions were announced after three separate meetings on extravasations (2008, 2009, and 2019) (3, 74, 75). The ACMUI position in all three cases—extravasations should remain exempted from reporting. However, a detailed review of the transcripts of these meetings reveal that some members understood the patient safety implications, but still recommended retaining the exemption. One ACMUI member when discussing an absorbed dose to tissue of 3–5 Gy (exceeding the 0.5 Sv reporting limit between 6 and 10 times) stated the following: “However, the first thing before us is, should NRC consider it as a medical event. Now if we consider this as a medical event, if we go through all the procedures and identify whatever- 3 or 4 or 5 - the patient will have to be informed; the physician have to be informed, blah blah blah *[sic]*, and then - you have to go into all the reporting mechanisms. And therefore, I am thoroughly against this being reported as a medical event” (76).

### Why Review These Hypotheses Now?

As a result of the petition, the NRC is considering evidence on whether diagnostic extravasations can exceed reporting limits. Current NRC reporting requirements in the United States state that a misadministration that results in a dose equivalent to tissue that exceeds 0.5 Sv (over 500× the dose the patient tissue receives during a proper administration) is indicative that an authorized user may be having issues handling radioactive material. Today, if a radiopharmaceutical is spilled on a patient and exposes patient tissue to >0.5 Sv, it is reportable.^4^ However, if an extravasation results in a tissue dose >0.5 Sv or significantly higher, this is NOT a reportable event and the patient is not required to be informed. Additionally, in Europe there is a new focus on safety and quality regarding diagnostic ionizing radiation. In the European Union, the Commission Staff Working Document on a Strategic Agenda for Medical Ionising Radiation Applications (SAMIRA) has a dedicated effort focused on “Quality and Safety of Medical Radiation Applications” (77). The Commission's objectives are to ensure “that citizens receive the best possible protection from the carcinogenic effects of ionizing radiation, while fully benefiting from the advantages it offers in battling cancer and other diseases. Notwithstanding recent developments in the European regulatory framework, there remains significant room for improvement of the quality and safety of medical radiation applications” (77). The Commission goes on to state that they intend “to launch a European Initiative on quality and safety aiming to ensure that the main diagnostic and therapeutic applications of ionizing radiation in Member States operate in line with high standards for quality and safety, in the interest of patients.”

Another important reason to address the commonly held hypotheses now, is the rapidly growing and important field of theranostics.^5^ As new therapeutic radiopharmaceuticals prove effective and are approved, administration volume will grow rapidly. This growing volume will result in more and more technologists administering these higher activity therapeutics. As a result, extravasations of therapies could increase. During the initial 44 administrations of Lutathera at a major US medical facility, 6 patients were extravasated (13.6%).^6^ Additionally, it has never been easier to characterize extravasations than today. A recent article describes using patient-specific biological clearance and reference tissue volumes (5cc) to quickly assess absorbed dose to tissue (7).

### Benefits of the New Hypotheses

We believe that once centers recognize that diagnostic radiopharmaceuticals extravasations can matter to patients and can be more easily characterized, patient care will improve, and inadvertent irradiation and wasteful healthcare spending may be reduced. Our findings from the literature and the five clinical case studies presented in this study support our belief. Patient A's lesion SUV_max_ was understated by 59% due to extravasation. Using the underestimation for comparison with a follow-up study could contribute to an incorrect interpretation of the treatment response. For example, a properly administered injection in a follow-up study could produce a lesion SUV higher than the Day 0 extravasated baseline SUV. The increase in SUV could be interpreted as stable or progressive disease when the proper interpretation may be partial response. Patient B's second restaging results appear to indicate a significant treatment improvement as compared to their first re-staging results; however, results from repeating the extravasated second re-staging procedure suggest stable disease. Patient C, like Patients A and B, required additional imaging and subsequent radiation exposure due to injection extravasation. Patient D showed lesions in the repeat scan that were not readily visible in the initial extravasated study. This may suggest that the extravasation resulted in limited bioavailability of the tracer over time causing a marked change in uptake for that patient. Patient E also showed significant differences in biodistribution between the initial and repeat imaging studies.

As precision medicine and quantification continues to become more important in the future, as ALARA principles grow more important, and as lower radiation doses are used in imaging, it is critical to reduce the frequency and severity of extravasation. Patient care and the field of nuclear medicine will benefit by reducing these misadministrations. While some questions remain unanswered about extravasations and while reporting of significant extravasations will involve some additional burden until extravasation rates are reduced, the recent advances in monitoring, detecting, and performing dosimetry justify this additional work to maximize the safety of our patients.

There were limitations to our work. Based on language skills and familiarity with the EV and FAERS databases, we only accessed two surveillance system databases. While they are a valuable source of information, surveillance systems do have limitations. By searching for certain terms, we only captured a sample of the available data. Our searches were also limited to information from submitted reports, likely due to the same conservative attitude toward reporting of extravasations noted by van der Pol et al. (20). The data available through these searches may comprise only a part of the surveillance data and do not necessarily confirm a causal relationship between the radiopharmaceutical and the reported adverse event. With respect to our local patient data, in our attempts to assess the impact of extravasations on patient management, efforts were made to minimize imaging parameter differences between procedures. While significant efforts were taken to ensure consistency of equipment and biological factors between scans, these patients were not part of a rigorous test-retest imaging protocol. As a result, some of the quantification differences may be attributable to factors other than extravasation. Additionally, because patient review was retrospective, we have no insight on possible adverse tissue and skin reactions. Future prospective work should include long-term patient follow-up to address radiation latent effects.

## Conclusions

Our findings suggest that significant extravasations can or have caused patient harm and can irradiate patients' tissue with doses that exceed medical event reporting limits and deterministic effect thresholds (78). In these cases, reports should be made, a patient follow-up plan should be devised, and the event should be analyzed with a goal toward reducing extravasations in the future. Further study is needed to fully assess the impact of significant nuclear medicine extravasations on patient imaging, dose to tissue, and possible patient harm.

## Data Availability Statement

The original contributions presented in the study are included in the article/supplementary material, further inquiries can be directed to the corresponding author/s.

## Ethics Statement

For Carilion Clinic, ethics committee approval was not necessary for this work. All participants consented to participation in the investigation. For UTMC the study was performed under an exempt protocol (IRB # 4607). All participants provided either written or telephone consent for their de-identified data to be used for teaching and publication purposes or were exempted from providing consent by the local institutional review board. Consent was documented in the patient files.

## Author Contributions

DO and JK contributed by obtaining, analyzing, interpreting patient data, and preparation of the manuscript. RL contributed to data analysis, interpretation, and preparation of the manuscript. JK contributed to data analysis, interpretation, and algorithm development. TB contributed to data collection, data analysis, interpretation, and preparation of the manuscript. IB contributed to data analysis and preparation of the manuscript. YF contributed by analyzing, interpreting patient data, and preparation of the manuscript. All authors contributed to manuscript review and editing and have read and approved the final manuscript.

## Conflict of Interest

RL, JK, TB, and IB are employed by the company Lucerno Dynamics LLC, the manufacturer of the Lara System described in this manuscript. The remaining authors declare that the research was conducted in the absence of any commercial or financial relationships that could be construed as a potential conflict of interest.

## Footnotes

^1^Available online at: https://www.regulations.gov/comment/NRC-2020-0141-0444 (accessed March 1, 2021).

^2^Available online at: https://www.regulations.gov/comment/NRC-2020-0141-0428 (accessed March 1, 2021).

^3^Available online at: https://www.regulations.gov/document/NRC-2020-0141-0137 (accessed March 1, 2021).

^4^NRC Event Number 53434. Available online at: https://www.nrc.gov/reading-rm/doc-collections/event-status/event/2018/20180611en.html (accessed March 1, 2021).

^5^Available online at: https://www.globenewswire.com/news-release/2020/10/26/2114163/0/en/Global-Theranostics-Markets-Report-2020-Oncology-Segment-Market-for-Theranostics-Should-Grow-from-73-1-Billion-in-2020-to-119-7-Billion-by-2025.html (accessed March 1, 2021).

^6^Available online at: https://ons.confex.com/ons/2019/cp/eposter.cgi?eposterid=442 (accessed March 1, 2021).

## Abbreviations

ACMUI: Advisory Committee on the Medical Use of Isotopes
EEA: European Economic Area
EV: EudraVigilance
FAERS: FDA's Adverse Event Reporting System
FDA: Food and Drug Administration
FDG: Fluorodeoxyglucose
Gy: Gray
IRB: Institutional Review Board
MDP: Methylene diphosphonate
MIP: Maximum intensity projection
mSv: Millisievert
NRC: Nuclear Regulatory Commission
PET/CT: Positron Emission Tomography/Computed Tomography
rem: Roentgen equivalent man
SPECT: Single Photon Emission Computed Tomography
SUV: Standardized Uptake Value
Sv: Sievert
TAC: Time-activity curve.
